# The effects of rain and flooding on leptospirosis incidence in sheep and cattle in New Zealand

**DOI:** 10.1080/00480169.2025.2540324

**Published:** 2025-08-12

**Authors:** E Sadler, E Vallee, J Watts, M Wada

**Affiliations:** a EpiCentre, Tāwharau Ora – School of Veterinary Science, Massey University, Palmerston North, New Zealand; b Biosecurity Surveillance (Animal Health), Ministry for Primary Industries, Wellington, New Zealand

**Keywords:** Leptospirosis, rainfall, climate change, flooding, livestock, zoonosis

## Abstract

**Aims:**

To describe the spatio-temporal patterns of leptospirosis case counts in sheep and cattle in New Zealand, and to assess their association with climate variables indicative of flooding and surface runoff. As livestock are a major reservoir of *Leptospira* spp. and an important source of zoonotic transmission, understanding these patterns is critical for informing livestock and public health interventions in the context of climate change.

**Methods:**

Confirmed cases of bovine and ovine leptospirosis from January 2011 to December 2023 were extracted from the Ministry for Primary Industries’ Animal Health Surveillance programme. Climate data was sourced from the National Institute of Water and Atmospheric Research. Using the χ^2^ test and Poisson regression models, the association between district-level case counts and four climate indices were examined: seasonal mean rainfall, seasonal frequency of extreme rainfall, seasonal mean soil moisture, and seasonal frequency of estimated surface runoff.

**Results:**

Findings indicated an average of 13 confirmed cases for sheep annually, with notable surges in 2017 (34 cases) and 2023 (36 cases), aligning with extreme climate events. Poisson regression models for sheep leptospirosis identified significant associations with extreme rainfall (incidence risk ratio (IRR) = 5.03; 95% CI = 1.18–21.45), mean rainfall (IRR = 1.25; 95% CI = 1.15–1.36), surface runoff (IRR = 1.09; 95% CI = 1.04–1.15), and soil moisture (IRR = 1.03; 95% CI = 1.02–1.03). Cattle leptospirosis was positively associated with surface runoff (IRR = 1.06; 95% CI = 1.02–1.10) and soil moisture (IRR = 1.01; 95% CI = 1.00–1.01). Associations with extreme rainfall (IRR = 1.46; 95% CI = 0.49–4.31) and mean rainfall (IRR = 1.07; 95% CI = 1.00–1.14) were not statistically significant.

**Conclusions:**

The outcomes of this study provide new evidence linking extreme rainfall, surface runoff, and other climate variables with increased leptospirosis case counts in sheep, with less pronounced but notable associations in cattle. These findings highlight the vulnerability of livestock to climate-driven disease pressures and suggest that future extreme weather events may increase the risk of leptospirosis outbreaks. This has important implications for targeted vaccination, surveillance, and public health preparedness in flood-prone rural regions of New Zealand.

## Abbreviations

ICC: Intra-class correlation coefficient
IRR: Incidence risk ratio
MPI: Ministry for Primary Industries
NIWA: National Institute of Water and Atmospheric Research

## Introduction

Climate change, characterised globally by elevated temperatures and shifting rainfall patterns (IPCC 2021), is a major driver of altered disease dynamics, influencing pathogen survival and transmission (McIntyre 2017; Mora *et al*. 2022). In New Zealand, recent climate trends show a measurable rise in average temperature and more frequent extreme weather events, including intense rainfall and flooding (Ministry for the Environment 2022; NIWA 2023). These changes were especially evident in the North Island in 2023, where recurring storms and cyclones caused significant disruption (Jones *et al*. 2023).

Cyclone Gabrielle in 2023 resulted in heavy rainfall, with extensive flooding across agricultural regions including Hawke’s Bay (Harrington *et al*. 2023). These conditions were followed by a five-fold rise in cases of human leptospirosis, possibly due to contact with contaminated floodwaters on livestock farms (ESR 2023). Extreme rainfall and flooding create favourable conditions for leptospire survival by increasing soil moisture and standing water (Chen *et al*. 2012). Occupational exposure among livestock workers also rises during wet periods, as shown in previous New Zealand studies (Dorjee *et al*. 2008). Internationally, similar associations between rainfall and leptospirosis in humans have been reported in Colombia, Argentina and Indonesia (Gutiérrez and Martínez-Vega 2018; López *et al*. 2019; Dhewantara *et al*. 2022). However, research on climate-driven changes influencing leptospirosis in New Zealand livestock remains limited.

Leptospirosis is a zoonotic disease caused by *Leptospira* spp., which can persist in the environment under conditions of high rainfall and warm temperatures (Cucchi *et al*. 2019). These conditions enhance *Leptospira* spp. survival and environmental contamination, increasing exposure risk. Rising temperatures may also expand the geographic range and seasonal persistence of infection, especially in temperate regions like New Zealand. Increased rainfall, especially extreme events, promotes runoff and flooding, spreading *Leptospira* via contaminated water and pasture (Chen *et al*. 2012; Mwachui *et al*. 2015). These climate shifts are likely to increase transmission risk and broaden leptospirosis in livestock systems.

Wildlife reservoirs, particularly rodents, are another significant risk factor for the spread of leptospirosis (Garba *et al*. 2018). In New Zealand, rats (*Rattus* spp.) and mice (*Mus musculus*) are known maintenance hosts for *Leptospira borgpetersenii* serovar Ballum, which is increasingly linked to human infections (Nisa *et al*. 2020). Other species such as hedgehogs (*Erinaceus europaeus*), possums (*Trichosurus vulpecula*) and deer (*Elaphus* spp.) have also been identified as carriers (Yupiana *et al*. 2019) and may contribute to environmental contamination alongside livestock. These overlapping reservoirs highlight the complex ecology of leptospirosis and support the need for an integrated One Health approach to surveillance and control.

In New Zealand, the primary livestock-associated serovar is *Leptospira borgpetersenii* serovar Hardjo, which is maintained in cattle and possibly sheep populations (Vallée *et al*. 2018). National surveillance data from 2009 to 2010 showed over 90% of sheep flocks and beef cattle herds had at least one animal seropositive for Hardjo (Dreyfus *et al*. 2018). Other endemic serovars, including Pomona, Tarassovi and Ballum, occur less frequently but have been associated with clinical disease (Sanhueza *et al*. 2013; Ridler *et al*. 2015).

The incidence of human leptospirosis is relatively high in New Zealand compared to other developed countries (El-Tras *et al*. 2018). Human leptospirosis in New Zealand has been traditionally associated with rural areas and occupations such as farming, where contact with animal urine, particularly from infected rodents or livestock, poses a risk (Goarant 2016). However, a more diverse picture with an increase in cases in the general population and of non-livestock-associated serovar Ballum is reported (Nisa *et al*. 2020). Seasonal outbreaks of leptospirosis associated with rainfall and floods have been reported worldwide, including in South America, Asia and Australia (Levett 2001; Chen *et al*. 2012; Coelho and Massad 2012).

Climate-related public health risks in New Zealand are often magnified following periods of heavy precipitation and flooding (Beggs *et al*. 2022). Understanding how environmental conditions influence the dynamics of leptospirosis in livestock is essential for guiding prevention strategies and mitigating human health risks. Sheep and cattle are the most abundant livestock in New Zealand, with an estimated 25 million sheep and 10 million cattle nationwide (Beef + Lamb New Zealand 2022). As known reservoirs of *Leptospira* spp., particularly serovar Hardjo, these species play a key role in zoonotic transmission. Despite this, quantitative information on how climate influences leptospirosis in livestock remains limited, largely due to diagnostic challenges and a lack of longitudinal data. To address this gap, the present study aimed to characterise spatial and temporal patterns of leptospirosis in sheep and cattle, and to measure the associations with flooding and surface runoff indices, using national laboratory diagnostic data from 2011 to 2023, integrated with climate data.

## Materials and methods

### Data source

#### Laboratory submission data

Laboratory submission data were obtained from the Ministry for Primary Industries (MPI) Animal Health Surveillance programme, which collects passive surveillance data from commercial veterinary laboratories across New Zealand. These records include submissions by practising veterinarians and contain anonymised information such as date, district, species and diagnosis. For this study, all sheep and cattle submissions resulting in a diagnosis of any *Leptospira* species or serovar between 1 January 2011 and 31 December 2023 were extracted. The leptospirosis case counts included all serologically confirmed or PCR-positive cases of leptospirosis, including all serovar types.

The data were initially processed into two separate datasets for sheep and cattle, counting leptospirosis cases per district per month. The cattle data encompassed both dairy and beef cattle populations. The leptospirosis case count data were aggregated temporally (month, season, year) or spatially (district, region, and North *vs* South Island) at the appropriate scale for further analyses. The seasons were classified as New Zealand spring (September, October and November), summer (December, January and February), autumn (March, April and May) and winter (June, July and August).

#### Climate data

Daily rainfall and daily soil moisture data were obtained from the National Institute for Water and Atmospheric Research’s (NIWA) Virtual Climate Station Network, which provides interpolated daily climate estimates across a 5 × 5 km grid using data from 150 automatic stations nationwide (Tait *et al*. 2006; NIWA 2017). Daily soil moisture (mm) was estimated by NIWA based on the daily rainfall and potential evapotranspiration, assuming fixed available water capacity.

Based on these variables, four indices were computed at the district level: seasonal mean rainfall, seasonal frequency of extreme rainfall, seasonal mean soil moisture, and seasonal frequency of surface runoff. Seasonal mean rainfall (mm) and seasonal mean soil moisture (mm) were calculated as mean daily inputs averaged across each 3-month season and district. The seasonal frequency of extreme rainfall was calculated as the district mean number of days per season when daily rainfall exceeded 100 mm, which was the threshold to describe extreme rainfall events (NIWA 2017). The seasonal frequency of surface runoff was calculated as the number of days per season when daily soil moisture was > 0 mm, indicating the soil store is at capacity and excess water is pooling at the surface.

#### Animal population data

Population data were obtained from the Agricultural Production Surveys and Censuses for 2017 undertaken by Statistics New Zealand (Stats NZ 2023). The data recorded the number of farms for sheep, dairy cattle, and beef cattle for each territorial authority in New Zealand.

### Statistical analysis

#### Case series

Descriptive and statistical analysis of the data were conducted using R version 4.2.2 (R Foundation for Statistical Computing, Vienna, Austria). The ggplot2 package was used to visualise the distribution of case counts across different species, months and years, as well as the regional- and district-level variations. Furthermore, we summarised the predominant presenting signs observed in both sheep and cattle, and serovars for cases where serovar results were available. We decided not to separate case counts by serovar type in the following analysis because of the incomplete nature of serovar testing as submitting veterinarians only requested testing for the serovar that they assumed to be the most prevalent. We used the confirmed leptospirosis case data to create choropleth maps of leptospirosis incidence rates per 1,000 farms per year.

#### Associations with climate variables

We used χ^2^ tests to investigate the crude association between the presence of leptospirosis cases and the four flood/runoff indices. The dataset was categorised into the explanatory variable and the response variable, which were both transformed into categorical variables. To account for potential delays between climate events and disease detection, case counts were aggregated across two consecutive seasons. The explanatory variable was the climate variable measured in the first of the two seasons used for aggregating case counts, to account for potential lag between exposure and detection. The climate variable was treated as a dichotomous variable (low/high), using the historical district mean as a threshold. The response variable was a binary outcome (yes/no) indicating whether at least one leptospirosis case was reported in the current or following season within each district. Regions with fewer reports were combined to enhance sample size. Additionally, we conducted individual χ^2^ tests at both the district and regional levels to assess associations with climate variables. This approach allowed us to evaluate the consistency in the observed patterns at different spatial resolutions, even when some geographical areas were not suitable for the analysis due to fewer leptospirosis cases.

Following the χ^2^ analysis, we fitted mixed-effects Poisson regression models to assess the association between climate indices and leptospirosis case counts. A Poisson distribution was selected after confirming no significant overdispersion when compared to a negative binomial model. For each species (sheep and cattle), four separate models were constructed to evaluate the effect of each climate index – seasonal frequency of extreme rainfall, seasonal mean rainfall, frequency of surface runoff, and mean soil moisture – on the number of confirmed leptospirosis cases in the two consecutive seasons. Climate variables were seasonal summary measurements on a continuous scale, corresponding to the first of the two seasons over which leptospirosis case counts were aggregated. District was included as a random effect. Fixed effects included season, island (North *vs*. South), and standardised (z-score standardised) counts of the number of farms (sheep farms for sheep models; beef and dairy farms for cattle), with variables retained if they met the p < 0.05 threshold in likelihood ratio testing. We standardised count of farms for numerical stability and included it as a candidate predictor. The outcome variable was defined as the count of confirmed leptospirosis cases per district over two consecutive seasons.

For each model, the intra-class correlation coefficient (ICC) was calculated, using the approximated approach proposed by Snijders and Bosker (2012):

ICC=var/(var+log(1+var))

where var is the variance of the random effects.

Additional models were run to test for interaction terms between climate variables and season. The significance of seasonal variations in the effects of climate variables on leptospirosis case counts was assessed by a likelihood test (*p* < 0.05). Season-specific effects of climate variables were estimated by re-running each model four times, each time changing the reference season.

To test whether associations persisted over a longer timeframe, we repeated the models using a combined leptospirosis case count over four consecutive seasons, while retaining climate data from the first season only to reflect the timing of exposure.

## Results

### Case series

Between 1 January 2011 and 31 December 2023, there were 156 laboratory-diagnosed cases of leptospirosis in sheep and 226 in cattle in New Zealand.

**Figure 1:**
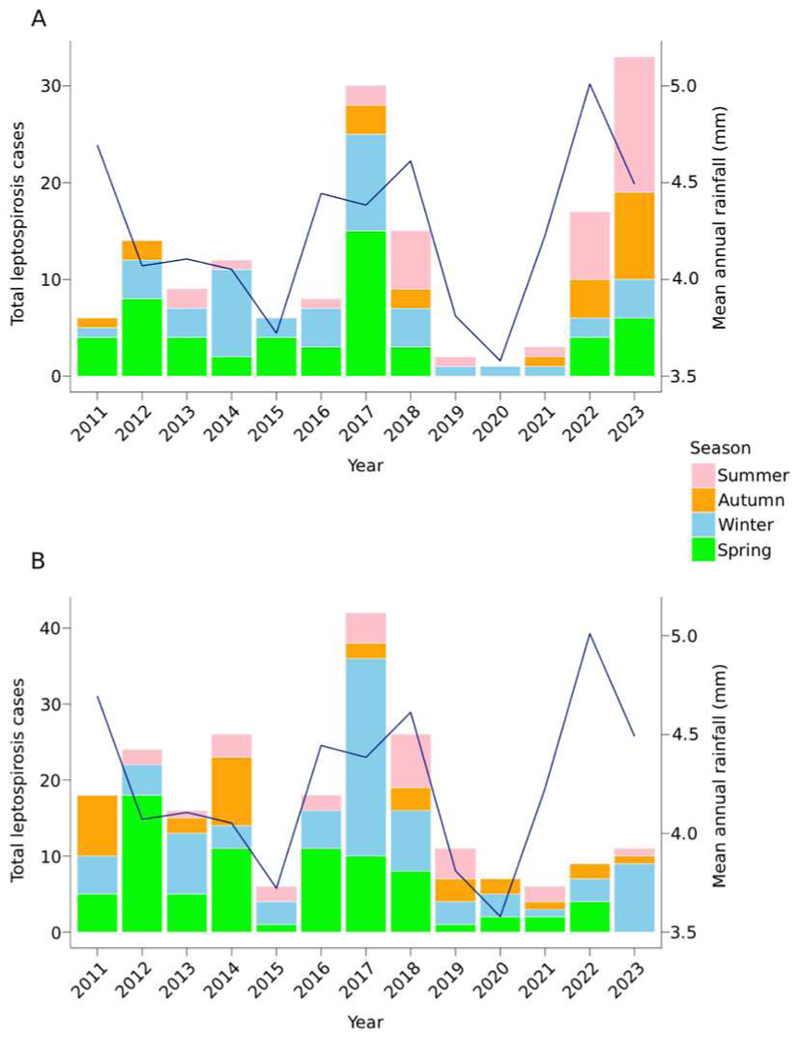
Seasonal counts of confirmed leptospirosis cases per year in (A) sheep and (B) cattle in New Zealand from January 2011 to December 2023 with annual mean rainfall (mm per day) represented by the blue line. For interpretation of the colour elements of this figure, please see the online version of the article.

In sheep, the highest proportion of confirmed cases occurred during spring (34% of cases), followed by winter (29%), summer (23%), and autumn (14%), with an average of 12.1 confirmed cases reported annually. The case count in 2023 was 33 cases, which was 2.8 times the historical average and the highest since 2011 (Figure 1(A)). This spike in 2023 was marked by an unusual seasonal pattern, with the majority of cases occurring in summer (n = 14; 42% of cases) and autumn (n = 9; 27%). There was a similar spike in cases in 2017 (n = 30), with most cases occurring in spring (n = 15; 50%) and winter (n = 10; 33%).

In cattle, the highest proportion of confirmed cases was reported in spring (37% of cases) and winter (36%), followed by autumn (14%) and summer (13%). Most (73%) cases in cattle were observed between June and November, including the main spring calving season (Figure 1(B)). The average annual count for confirmed cases in cattle was 17.5. In 2023, the number of cattle cases (n = 18) was similar and aligned with the typical seasonal pattern, with the highest counts observed during winter (n = 9; 53%) and spring (n = 5; 29%). A notable surge occurred in 2017 (n = 42), similar to the pattern seen in sheep, with the majority of cases occurring in winter (n = 26; 62%) and spring (n = 10; 24%).

**Figure 2:**
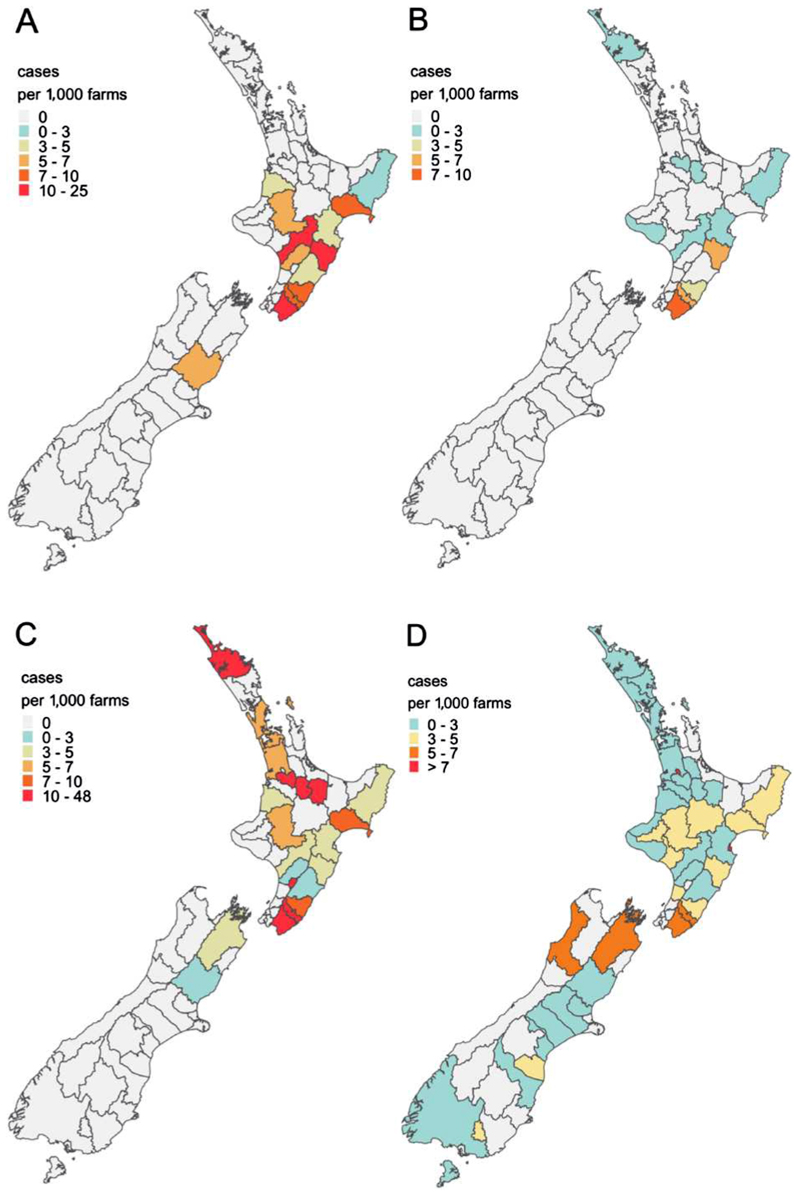
Incidence rates of confirmed leptospirosis cases per 1,000 farms per year for the districts of New Zealand. (A) The annual incidence rates in 2023 for sheep; and (B) the mean annual incidence rates from 2011 to 2023 for sheep; (C) the annual incidence rates in 2023 for cattle; and (D) the mean annual incidence rates from 2011 to 2023 for cattle. Incidence rates are expressed as cases per 1,000 farms per year, with farm population data derived from the 2017 agricultural census. For interpretation of the colour elements of this figure, please see the online version of the article.

Geographically, districts with higher incidence rates in sheep in 2023 were concentrated in the North Island, particularly along the east coast (Figure 2(A)). The highest district-level incidence rates occurred in South Wairarapa (24 cases per 1,000 farms per year) and Central Hawke’s Bay (23 cases per 1,000 farms per year). This distribution aligned with the spatial pattern of average annual incidence rate observed across the 2011–2023 period (Figure 2(B)). Conversely, confirmed cases in cattle in 2023 were almost exclusively reported in the North Island, particularly along the east coast (Figure 2(C)). This contrasts with historical patterns from 2011 to 2023, when cattle incidence rates were more evenly distributed across both the North and South Islands (Figure 2(D)).

Common presenting signs for confirmed cases of leptospirosis in sheep and cattle are presented in Table 1 and the serovars isolated in Table 2.

### Association with climate variables

#### χ^2^ analysis

In Manawatū-Whanganui, Hawke’s Bay and Waikato, the presence of sheep leptospirosis over two seasons was significantly associated with three climate variables: seasonal mean rainfall, surface runoff frequency, and soil moisture (Table 3), while no significant association was found with the frequency of extreme rainfall events. The Wellington region had a significant association between sheep leptospirosis and higher frequency of surface runoff. The Bay of Plenty and Gisborne combination, adjacent to Hawke’s Bay, showed significant associations between sheep leptospirosis and frequency of extreme rainfall, frequency of surface runoff and soil moisture. The remaining regions in New Zealand did not exhibit a significant association with any of the climate variables.

In the Manawatū-Whanganui region, cattle leptospirosis was significantly associated with all four climate variables studied, while the Hawke’s Bay region did not exhibit any significant associations (Table 4). In the neighbouring regions, Waikato and Wellington, a higher frequency of surface runoff was associated with the presence of leptospirosis over the next two seasons. In addition, Wellington had a significant association between cattle leptospirosis and higher seasonal mean rainfall. Similar to the observations in sheep, the combined regions of Bay of Plenty and Gisborne had significant associations between cattle leptospirosis and frequency of extreme rainfall, frequency of surface runoff, and soil moisture. The remaining regions in New Zealand did not exhibit a significant association with any of the climate variables.

Amalgamating regions within each island, there was a significant association in both sheep and cattle between higher frequency of surface runoff or higher seasonal mean soil moisture and the presence of leptospirosis over two consecutive seasons in the North Island (Table 5). Additionally, for sheep in the North Island, an association was observed between the presence of leptospirosis and the frequency of extreme rainfall. The count of diagnosed cases in sheep populations reported in the South Island was too low (n = 9) and not suitable for χ^2^ analysis. The cattle population on the South Island showed an association between presence of leptospirosis over the following two seasons and higher frequency of extreme rainfall and higher frequency of surface runoff.

#### Poisson regression models (sheep)

The results from the four-season analysis are included in Supplementary Information and were mostly consistent with the two-season analysis presented here.

**Figure 3:**
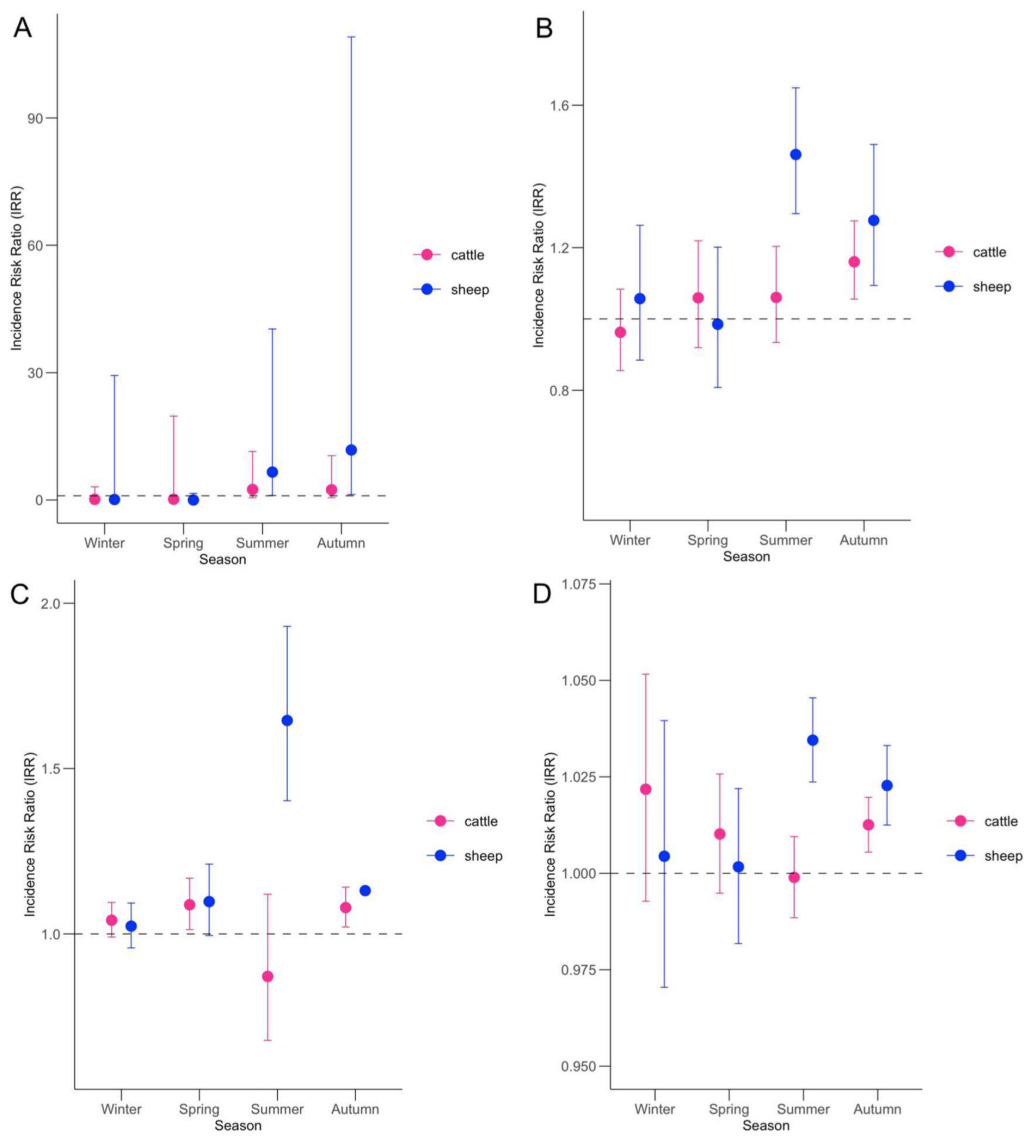
The interaction between incidence risk ratio and season for sheep and cattle for (A) frequency of extreme rainfall; (B) seasonal mean rainfall; (C) frequency of surface runoff; and (D) soil moisture over two consecutive seasons. The interaction between climate variable and season was statistically significant for sheep, but not for cattle. For interpretation of the colour elements of this figure, please see the online version of the article.

As a crude baseline assessment, models without an interaction term are presented (Table 6). These results indicated that all four climate variables averaged across four seasons had a significant positive association with the number of diagnosed sheep leptospirosis cases observed across two consecutive seasons. Within a given district, an increase in the frequency of extreme rainfall by 1 day per season was associated with a 5.03-fold (95% CI = 1.18–21.45) increase in the risk of reporting leptospirosis over the subsequent two seasons (Table 6). Additionally, within a given district, a 1 mm increase in seasonal mean rainfall was associated with a 1.25-fold (95% CI = 1.15–1.36) increase in the risk of reporting leptospirosis over the next two seasons relative to the district’s baseline level. Further analyses suggested significant interactions between the four climate variables and season (frequency of extreme rainfall: *p* < 0.01; seasonal mean rainfall: *p* < 0.001; frequency of surface runoff: *p* < 0.001; and seasonal mean soil moisture: *p* < 0.05), indicating that the association between the climate variables and the count of sheep leptospirosis cases varied by season of climate event occurrence. The estimated season-specific incidence risk ratios (IRR) for the four climate variables are shown in Figure 3. Within a given district, associations between climate variables and sheep leptospirosis were significantly positive only when climate events occurred in summer (IRR extreme rainfall 6.58 (95% CI = 1.07–40.31); mean rainfall 1.46 (95% CI = 1.30–1.65); runoff 1.65 (95% CI = 1.40–1.93); mean soil moisture 1.03 (95% CI = 1.02–1.05)) or autumn (IRR extreme rainfall 11.78 (95% CI = 1.27–109.11), mean rainfall 1.28 (95% CI = 1.09–1.49), runoff 1.13 (95% CI = 1.13–1.13), mean soil moisture 1.02 (95% CI = 1.01–1.03)). In contrast, there was no significant associations between climate variables in winter and spring and sheep leptospirosis case counts.

The SD of the district-level random intercepts ranged from 2.34 to 2.48. The corresponding ICC values ranged from 0.745 to 0.758, indicating that a substantial proportion of the variance in sheep leptospirosis case counts was attributable to differences between districts.

#### Poisson regression models (cattle)

The risk of cattle leptospirosis was positively associated with the frequency of surface runoff (IRR = 1.06; 95% CI = 1.02–1.10) and seasonal mean soil moisture (IRR = 1.01; 95% CI = 1.00–1.01), whereas there was no significant association with the frequency of extreme rainfall (IRR = 1.46; 95% CI = 0.49–4.31) and seasonal mean rainfall (IRR = 1.07; 95% CI = 1.00–1.14). Within a given district, an increase in the frequency of surface runoff by 1 day per season was associated with a 1.06-fold increase in the risk of reporting leptospirosis over the following two seasons (Table 7).

Interaction was not significant for cattle leptospirosis, indicating that while surface runoff and soil moisture had significant associations with disease risk, their effects did not vary by season.

Similar to the sheep models, the SD of the district-level random intercepts ranged from 1.41 to 1.43, with the ICC values ranging from 0.646 to 0.648, indicating that a substantial proportion of the variance in leptospirosis case counts was attributable to differences between districts.

## Discussion

This study analysed the number of confirmed leptospirosis cases in sheep and cattle in relation to climate factors in New Zealand, using laboratory submission data from 2011 to 2023, demonstrating positive associations between seasonal frequency of extreme rainfall, seasonal frequency of runoff, seasonal mean rainfall, seasonal mean soil moisture and leptospirosis incidence in sheep. For cattle, seasonal frequency of runoff and seasonal mean soil moisture were significantly associated with leptospirosis incidence. To the best of our knowledge, this is the first study to demonstrate the association between climate variables and leptospirosis in animals in New Zealand.

Our study demonstrated that, on average, an increase in the frequency of extreme rainfall by 1 day per season was associated with a five-fold increase in leptospirosis reporting in sheep over the subsequent two seasons, with the magnitude of the effects varying by season. Increases in surface runoff also significantly raised the risk of leptospirosis in sheep, with the effects varying by season. These results were in line with the χ^2^ analysis, with association with extreme rainfall and surface runoff being the most frequently detected over the country, particularly in regions with higher leptospirosis reports, such as Manawatū-Whanganui, Hawke’s Bay, and Waikato. This suggests that extreme rainfall and subsequent flooding are the main climatic drivers for ovine leptospirosis outbreaks, which is supported by the two peaks observed in 2017 and 2023 when large flooding events were observed in the North Island. The high ICC values, particularly in the sheep models (0.75–0.76), suggest strong spatial clustering of leptospirosis cases at the district level and support the inclusion of district-level random effects to account for this structure. Recent advances in understanding the environmental factors affecting survival of *Leptospira* spp. indicate that leptospires survive in soil, and heavy rainfall and flooding re-suspend and mobilise *Leptospira*-contaminated soil particles (Bierque *et al*. 2020; Thibeaux *et al*. 2024). The models also identified seasonal mean rainfall and soil moisture as associated with an increased risk of leptospirosis reporting, indicating that climate change and long-term trend changes in rainfall will affect the incidence of ovine leptospirosis. This aligns with findings in human leptospirosis, where increased rainfall and flooding have similarly been associated with elevated case numbers both overseas (Douchet *et al*. 2022) and in New Zealand (Tana 2021). These wet weather events are a recognised risk factor for human leptospirosis infection, as they lead to an increase in environmental exposure, or contact with infected livestock or contaminated floodwater (Dreyfus *et al*. 2015). Previous livestock research has provided limited and indirect evidence suggesting that leptospirosis prevalence may increase following heavy rainfall in New Zealand. For instance, Dorjee *et al*. (2008) observed a higher prevalence of leptospirosis in lambs sampled at abattoirs after periods of significant flooding, compared to those sampled in the subsequent reproductive season.

Cattle showed increased leptospirosis risk following elevated surface runoff and soil moisture, both of which are influenced by rainfall and evapotranspiration interacting with soil type and other climate variables. These findings suggest that the generation of surface water, rather than rainfall alone, may be a key trigger for infection in cattle. In addition, no spike in bovine leptospirosis cases was observed in 2023. This may be due to the seasonal nature of clinical signs in cattle linked to the calving cycle, leading to cases being primarily detected in winter and spring. Due to a lack of data, we could not differentiate the leptospirosis incidence between beef cattle and dairy cattle; therefore, we used the combined cattle incidence. When beef population size and dairy population size were included in the Poisson regression model, a higher number of beef farms in a district was significantly associated with increased cattle leptospirosis case counts, whereas dairy farm numbers were not significantly associated. This pattern may reflect differences in management practices, such as the co-grazing of approximately 63% of sheep and 73% of beef cattle; differences in terrain, such as hill country *vs* flat land; or differences in vaccination status (Dreyfus *et al*. 2018). In New Zealand, most dairy cattle are vaccinated against Hardjo and Pomona, reducing the likelihood of shedding (Yupiana *et al*. 2020), while vaccination rates in sheep flocks and beef herds are much lower (Dreyfus *et al*. 2018). These factors may help explain the differences observed in cattle leptospirosis patterns across farm types.

We found a notable difference in the presenting signs of leptospirosis cases between sheep and cattle, which may help to explain differences in the timing of detection and reporting between seasons. In sheep, commonly reported clinical signs included being found dead, abortion/stillbirth, urinary disease, and jaundice. In contrast, 96% of confirmed cattle cases presented with abortion or stillbirth – signs that are more likely to be recognised and investigated before and during the spring calving season, when reproductive losses are closely monitored. This may have contributed to a seasonal imbalance in the timing of reported cattle cases. In New Zealand, a study found that *Leptospira* spp. accounted for 8.3% of all cattle abortions (Sanhueza *et al*. 2013). Similar studies in Europe have also reported abortion as the most common presenting sign of leptospirosis in cattle, followed by other reproductive abnormalities (Sohm *et al*. 2023).

Due to the limitations of passive surveillance and potential delays between exposure and diagnosis, we analysed case counts over two consecutive seasons to capture both immediate and delayed effects of climate events. Leptospirosis has an incubation period of 7–14 days, extending up to 30 days in some cases, and leptospires may survive in moist environments for weeks to months (Dirar 2016). Passive surveillance data likely underestimate the true incidence by excluding unconfirmed, subclinical or undiagnosed cases, which may weaken observed associations. It also lacks contextual data, limiting control for confounding variables. Additionally, the climate data used were not specific to farmland, so some flooding events may not have aligned with live-stock exposure.

Another limitation of our study was the lack of comprehensive data on *Leptospira* serovars. Our data only indicate the presence of the particular serovars that were tested for and confirmed as positive. However, not all cases were tested for serovars, and even when testing was performed, it was usually limited to suspected serovars, resulting in a dominance of the common serovars and potential biases. Further testing to determine the serovars present would be useful as different *Leptospira* spp. vary in their ability to survive in the environment (Bulach *et al*. 2006).

Widespread vaccination not only protects dairy herds from clinical disease but also significantly reduces environmental shedding of leptospires, lowering the risk of transmission to unvaccinated animals and humans (Ellis 2015). In occupational settings, such as dairy farming and abattoir work, reduced bacterial shedding translates into lower exposure risk for workers, contributing to improved occupational health outcomes (Grout *et al*. 2020). At a broader scale, this supports the development of herd immunity within livestock populations, which is a critical component of integrated One Health strategies to control zoonotic diseases (World Health Organization 2011).

Diagnoses were less frequent in the South Island compared with the North Island for both sheep and cattle (sheep IRR = 0.01, cattle IRR = 0.22). While previous studies reported high seroprevalence in southern regions (Dreyfus *et al*. 2018), our findings likely reflect differences in study design, time period, and passive reporting. Variability in veterinary access, farm practices, or reporting behaviour may also contribute to under-detection in some regions.

Rainfall events indicate an increased risk for public health, as rising leptospirosis incidence in livestock elevates the risk of transmission to humans. Livestock populations act as reservoirs, maintaining and shedding the bacteria into the environment. Farmers and occupational workers are particularly at risk due to close contact with animals and contaminated environments (Dreyfus *et al*. 2018). Understanding the effects of climate change on leptospirosis in livestock populations is critical for effective disease management and strategies to protect public health.

The immediate effects of wet weather events on sheep leptospirosis varied by season, with more pronounced effects in summer and autumn. For cattle leptospirosis, the effect of rainfall was not significant across seasons, whereas the effects of soil moisture and surface runoff were significant and consistent across seasons. This may reflect a small effect size, limited study power, or both, for the climate impacts on cattle leptospirosis. It is also possible that relevant climate variables, such as temperature and soil type, interact differently across livestock species, as suggested by Douchet *et al*. (2022) and Davignon *et al*. (2023).

This study demonstrated that four climate variables related to flooding and runoff were significantly associated with increased leptospirosis risk in sheep, whereas associations with soil moisture and surface runoff were observed in cattle. These findings suggest species-specific differences in climate vulnerability, potentially influenced by production systems or regional factors. A One Health approach, integrating human, animal and environmental health perspectives, is necessary for monitoring and managing leptospirosis. Understanding these dynamics is essential for improving livestock disease surveillance and anticipating climate-related outbreaks. As extreme weather events become more frequent, integrating climate data into animal health monitoring will be increasingly important for targeted, evidence-based response strategies.

## Supplementary Material

Suppl 1 Supplemental data for this article can be accessed online at https://doi.org/10.1080/00480169.2025.2540324.

## Figures and Tables

**Table 1:** Number (percentage) of sheep and cattle diagnosed with leptospirosis between January 2011 and December 2023 in New Zealand presenting with typical signs of leptospirosis.

Sheep (*n =* 156)			Cattle (*n* = 226)	
Presenting signs	Number (%)		Presenting signs	Number (%)
Death – found dead	104 (66.6%)		Abortion/stillbirth	217 (96%)
Abortion/stillbirth	68 (43.5%)		Death – found dead	51 (22.5%)
Urinary disease	42 (26.9%)		Urinary disease	44 (19.5%)
Jaundice	38 (24.3%)		Ill thrift/emaciation/weight loss	33 (14.6%)
Ill thrift/emaciation/weight loss	33 (21%)		Abnormalities of the reproductive tract	29 (12.8%)
Abnormalities of the reproductive tract	21 (13.5%)		Anorexia	15 (6.6%)
Depression/weakness	19 (12.2%)		Infertility	15 (6.6%)
Infertility	16 (10.2%)		Anaemia	12 (5.3%)
Respiratory signs (unspecified)	13 (8.3%)		Alimentary (unspecified)	11 (4.9%)
Anorexia	10 (6.4%)		Depression/weakness	11 (4.9%)
Mass mortality	10 (6.4%)		Acute febrile disease	10 (4.4%)
Anaemia	9 (5.8%)		Production drop	10 (4.4%)
Acute febrile disease	7 (4.5%)		Metabolic disorder	7 (3.1%)
Diarrhoea/dysentery	7 (4.5%)		Nervous signs	7 (3.1%)
Death – embryonic	6 (3.8%)		Skin lesions (including colour change)	6 (2.6%)
Alimentary (unspecified)	5 (3.3)		Death – embryonic	4 (1.8%)
Lameness	3(1.9%)		Organs of special sense abnormalities	4 (1.8%)
Mastitis	2 (1.3%)		Diarrhoea/dysentery	3 (1.3%)
No history available	2 (1.3%)		Jaundice	3 (1.3%)
Perinatal loss	2 (1.3%)		Circulatory	2 (0.9%)
Production drop	2 (1.3%)		Congenital abnormalities	2 (0.9%)
Skin lesions (including colour change)	2 (1.3%)		Perinatal loss	2 (0.9%)
Circulatory	1 (0.7%)		Mastitis	1 (0.4%)
Nervous signs	1 (0.7%)		No clinical signs	1 (0.4%)

**Table 2:** Distribution of confirmed leptospirosis cases by serovar from New Zealand sheep and cattle suspected of infection with leptospirosis between 2011 and 2023. Percentages represent the proportion of total confirmed cases for each species.

Serovar	Sheep	Cattle
Hardjo	42 (27%)	76 (34%)
Pomona	61 (39%)	82 (36%)
Tarassovi	0 (0%)	9 (4%)
Unconfirmed	53 (34%)	59 (26%)
Total	156	226

**Table 3:** χ^2^ analysis of the relationship within the farms of a regions of New Zealand from 2011 to 2023 between whether at least one case of leptospirosis in sheep was reported in the current or following season (absent/present) and the number of occasions when the value of the climate variable in the first season was below (low) or above (high) the historical mean value.

Region	Leptospirosis in sheep^a^	Climate index																		
		Frequency of extreme rainfall (days/season)					Seasonal mean rainfall (mm)					Seasonal mean soil moisture deficit (mm)					Frequency of surface runoff (days/season)			
		Low	High	χ^2^	P-value		Low	High	χ^2^	P-value		Low	High	χ^2^	P-value		Low	High	χ^2^	P-value
Manawatū-Whanganui	Absent	264	38				170	132				139	163				227	75		
	Present	49	14				22	41				18	45				25	38		
				3.210	0.073				8.710	0.003				5.790	0.016				29.070	< 0.001
Hawke’s Bay	Absent	142	20				96	66				82	80				112	50		
	Present	35	11				17	29				10	36				22	24		
				2.920	0.087				6.310	0.012				10.970	0.001				6.200	0.013
Northland and Auckland	Absent	166	33				108	91				80	119				131	68		
	Present	7	2				5	4				4	5				5	4		
				0.000	1.000				0.000	1.000				0.000	1.000				0.080	0.783
Bay of Plenty and Gisborne	Absent	290	60				212	138				149	201				235	115		
	Present	7	7				4	10				5	9				3	11		
				7.610	0.006				4.460	0.035				0.050	0.815				10.490	0.001
Waikato	Absent	447	55				280	222				199	303				333	169		
	Present	16	2				5	13				2	16				6	12		
				0.000	1.000				4.430	0.035				4.820	0.028				6.950	0.008
Wellington	Absent	260	117				195	182				165	212				253	124		
	Present	26	13				23	16				12	27				11	28		
				0.010	0.910				0.480	0.487				1.940	0.164				21.420	< 0.001
Marlborough and Canterbury	Absent	404	159				314	249				259	304				375	188		
	Present	9	0				4	5				4	5				4	5		
				2.250	0.133				0.120	0.733				0.000	1.000				1.080	0.298

a At least one case of leptospirosis diagnosed in sheep in the current or following season.

**Table 4:** χ^2^ analysis of the relationship within the farms of a regions of New Zealand from 2011 to 2023 between whether at least one case of leptospirosis in cattle was reported in the current or following season (absent/present) and the number of occasions when the value of the climate variable in the first season was below (low) or above (high) the historical mean value.

Region	Leptospirosis in cattle^a^	Climate index																		
		Frequency of extreme rainfall (days/season)					Seasonal mean rainfall (mm)					Seasonal mean soil moisture deficit (mm)					Frequency of surface runoff (days/season)			
		Low	High	χ^2^	P-value		Low	High	χ^2^	P-value		Low	High	χ^2^	P-value		Low	High	χ^2^	P-value
Manawatū-Whanganui	Absent	270	36				173	133				142	164				230	76		
	Present	42	16				19	39				14	44				21	37		
				8.720	0.003				8.710	0.003				8.980	0.003				32.770	< 0.001
Hawke’s Bay	Absent	135	22				88	69				75	82				105	52		
	Present	42	9				25	26				17	34				29	22		
				0.170	0.684				0.510	0.475				2.690	0.101				1.280	0.259
Northland and Auckland	Absent	141	25				94	72				71	95				112	54		
	Present	32	10				19	23				13	29				24	18		
				1.260	0.261				1.320	0.250				1.480	0.223				1.160	0.282
Bay of Plenty and Gisborne	Absent	279	52				203	128				143	188				229	102		
	Present	18	15				13	20				11	22				9	24		
				15.750	< 0.001				5.110	0.024				0.830	0.363				21.470	< 0.001
Waikato	Absent	414	54				263	205				186	282				312	156		
	Present	49	3				22	30				15	37				27	25		
				1.060	0.303				3.110	0.078				1.910	0.167				3.860	0.050
Wellington	Absent	261	118				192	187				165	214				252	127		
	Present	25	12				26	11				12	25				12	25		
				0.000	1.000				4.440	0.035				1.280	0.259				15.430	< 0.001
Taranaki	Absent	97	38				66	69				61	74				76	59		
	Present	19	2				13	8				5	16				8	13		
				2.400	0.121				0.770	0.381				2.580	0.108				1.750	0.186
West Coast and Southland	Absent	103	47				80	70				43	107				98	52		
	Present	6	0				6	0				0	6				2	4		
				1.410	0.235				3.370	0.066				1.160	0.282				1.360	0.243
Marlborough and Canterbury	Absent	398	156				307	247				256	298				369	185		
	Present	15	3				11	7				7	11				10	8		
				0.650	0.422				0.060	0.812				0.140	0.709				0.520	0.470

a At least one case of leptospirosis diagnosed in cattle in the current or following season.

**Table 5:** χ^2^ analysis of the relationship for sheep and cattle within the North and South Island of New Zealand from 2011 to 2023 between whether at least one case of leptospirosis was reported in the current or following season (absent/present) and the number of occasions when the value of the climate variable in the first season was below (low) or above (high) the historical mean value.

Species	Island	Leptospirosis Cases^a^	Climate index																		P-value
			Frequency of extreme rainfall (days/season)					Seasonal mean rainfall (mm)					Seasonal mean soil moisture deficit (mm)					Frequency of surface runoff (days/season)			
			Low	High	χ^2^	P-value		Low	High	χ^2^	P-value		Low	High	χ^2^	P-value		Low	High	χ^2^	
Sheep	North	Absent	1852	364				1240	976				955	1261				1339	877		
		Present	148	45				100	93				57	136				103	90		
					5.50	0.02				1.07	0.30				12.85	0.00				3.39	0.07
	South	Absent	1031	248				848	431				581	698				821	458		
		Present	9	0				8	1				4	5				5	4		
					1.09	0.30				1.16	0.28				0.00	1.00				0.04	0.85
Cattle	North	Absent	1727	341				1166	902				910	1158				1262	806		
		Present	236	61				152	145				91	206				148	149		
					2.74	0.10				2.64	0.10				18.46	< 0.001				13.06	< 0.001
	South	Absent	999	236				829	406				574	661				797	438		
		Present	19	11				18	12				7	23				14	16		
					4.68	0.03				0.39	0.53				5.42	0.019				3.32	0.07

a At least one case of leptospirosis diagnosed in cattle and sheep in the current or following season.

**Table 6:** Estimated^a^ incidence risk ratios (IRR) (95% CI) showing relative risk of leptospirosis in sheep in New Zealand across two consecutive seasons associated with climate indices.

Variable	Climate indices										
	Frequency of extreme rainfall			Seasonal mean rainfall			Frequency of surface runoff			Seasonal mean soil moisture	
	IRR (95% CI)	*P*-value		IRR (95% CI)	*P*-value		IRR (95% CI)	*P*-value		IRR (95% CI)	*P*-value
Intercept	–4.48	<0.001		–5.32	<0.001		–4.57	<0.001		–2.63,	<0.001
Climate variable	5.03 (1.18–21.45)	0.03		1.25 (1.15–1.36)	<0.001		1.09 (1.04, 1.15)	<0.001		1.03 (1.02, 1.03)	<0.001
Season											
Summer	Reference			Reference			Reference			Reference	
Autumn	1.15 (0.81–1.62)	0.44		1.01 (0.71–1.43)	0.96		0.87 (0.59–1.28)	0.47		0.47 (0.31–0.73)	<0.001
Winter	1.68 (1.21–2.33)	<0.001		1.22 (0.8–1.71)	0.24		0.6 (0.31–1.18)	0.14		0.25 (0.14–0.45)	<0.001
Spring	1.52 (1.08–2.13),	0.02		1.25 (0.89–1.74)	0.19		1.05 (0.72–1.53)	0.81		0.39 (0.24–0.63)	<0.001
Number of farms	6.85 (2.84–16.51)	<0.001		7.24 (2.9–18.1)	<0.001		6.77 (2.73–16.78)	<0.001		6.9 (2.76–17.24)	<0.001
Island											
North	Reference			Reference			Reference			Reference	
South	0.01 (0–0.07)	<0.001		0.01 (0–0.07)	<0.001		0.01 (0–0.08)	<0.001		0.01 (0–0.08)	<0.001

a From Poisson regression models, accounting for season, number of farms, and North/South Island as fixed effects and district as a random effect.

**Table 7:** Estimated^a^ incidence risk ratios (IRR) (95% CI) showing relative risk of leptospirosis in cattle in New Zealand across two consecutive seasons associated with climate indices.

Variable	Climate indices										
	Frequency of extreme rainfall			Seasonal mean rainfall			Frequency of surface runoff			Seasonal mean soil moisture	
	IRR (95% CI)	*P*-value		IRR (95% CI)	*P*-value		IRR (95% CI)	*P*-value		IRR (95% CI)	*P*-value
Intercept	–3.38	<0.001		–3.59	<0.001		–3.44	<0.001		–2.68,	<0.001
Climate variable	1.46 (0.49–4.31)	0.49		1.07 (1–1.14)	0.06		1.06 (1.02–1.1)	<0.001		1.01 (1–1.01)	<0.001
Season											
Summer	Reference			Reference			Reference			Reference	
Autumn	1.87 (1.37–2.55)	<0.001		1.79 (1.3–2.44)	<0.001		1.55 (1.11–2.17)	0.01		1.39 (0.97–2)	0.07
Winter	2.55 (1.89–3.44)	<0.001		2.3 (1.69–3.14)	<0.001		1.29 (0.76–2.21)	0.35		1.28 (0.77–2.13)	0.35
Spring	1.77 (1.28–2.43)	<0.001		1.67 (1.22–2.29)	<0.001		1.41 (1–1.99)	0.05		1.08 (0.71–1.66)	0.71
Number of farms											
Dairy	1.1 (0.7–1.73)	0.68		1.08 (0.69–1.71)	0.73		1.04 (0.66–1.65)	0.86		1.07 (0.68–1.68)	0.78
Beef	1.93 (1.22–3.04)	<0.001		1.96 (1.24–3.1)	<0.001		2.02 (1.27–3.21)	<0.001		1.98 (1.26–3.13)	<0.001
Island											
North	Reference			Reference			Reference			Reference	
South	0.22 (0.09–0.54)	<0.001		0.22 (0.09–0.55)	<0.001		0.22 (0.09–0.54)	<0.001		0.23 (0.09–0.57)	<0.001

a From Poisson regression models, accounting for season, number of farms, and North/South Island as fixed effects and district as a random effect.
