# Supplementary material for: The effects of rain and flooding on leptospirosis incidence in sheep and cattle in New Zealand
Source: N Z Vet J. Author manuscript; Available in PMC 2026 May 1. (PMC7618971; doi:10.1080/00480169.2025.2540324)
Supplement: Suppl 1 — Supplemental data for this article can be accessed online at https://doi.org/10.1080/00480169.2025.2540324. [file EMS212927-supplement-Suppl_1.pdf]

The effects of rain and flooding on leptospirosis incidence in sheep and cattle in New Zealand

E Sadler[a], E Vallee[a], J Watts[b] and M Wada[a§]

[a] EpiCentre, Tāwharau Ora – School of Veterinary Science, Massey University, Palmerston North, New Zealand
[b] Biosecurity Surveillance (Animal Health), Ministry for Primary Industries, Wellington, New Zealand
[§] Author for correspondence: Email: M.Wada@massey.ac.nz

Table S1. The estimated incidence risk ratios (IRR) (95% confidence intervals) for climate indices on the combined number of leptospirosis cases in sheep across four consecutive seasons per district in New Zealand by Poisson regression models, accounting for season, number of farms, and North/South island as fixed effects and district as a random effect.

Fixed Effects		Climate indices			
		Frequency of extreme rainfall (days/season)	Seasonal mean rainfall (mm)	Frequency of surface runoff (days/season)	Seasonal mean soil moisture (mm)
Intercept	Intercept, p-value	−4.3, < 0.001	−4.88, < 0.001	−4.27, < 0.001	−3.49, < 0.001
Climate variable	IRR, p-value	3.97 (3.96, 3.97), < 0.001	1.15 (1.09, 1.23), < 0.001	1.06 (1.06, 1.06), < 0.001	1.01 (1.01, 1.02), < 0.001
Season: summer	IRR, p-value	Reference			
Season: autumn	IRR, p-value	0.94 (0.94, 0.94), < 0.001	0.88 (0.7, 1.11), 0.28	0.8 (0.8, 0.8), < 0.00	0.66 (0.5, 0.87), < 0.001
Season: winter	IRR, p-value	0.99 (0.99, 0.99), < 0.001	0.81 (0.64, 1.02), 0.07	0.5 (0.5, 0.5), < 0.001	0.41 (0.27, 0.62), < 0.001
Season: spring	IRR, p-value	1.04 (1.04, 1.04), < 0.001	0.91 (0.73, 1.14), 0.43	0.81 (0.81, 0.81), < 0.001	0.55 (0.4, 0.77), < 0.001

The content of this supplementary information has not been edited. All risk and liability rest with the authors.

Fixed Effects		Climate indices			
		Frequency of extreme rainfall (days/season)	Seasonal mean rainfall (mm)	Frequency of surface runoff (days/season)	Seasonal mean soil moisture (mm)
Farm count: sheep	IRR, p-value	9.79 (9.78, 9.81), < 0.001	10.91 (3.14, 37.82), < 0.001	10.5 (10.48, 10.51), < 0.001	10.6 (3.09, 36.35), < 0.001
Island: North	IRR, p-value	Reference			
Island: South	IRR, p-value	0 (0, 0), < 0.001	0 (0, 0.06), < 0.001	0 (0, 0), < 0.001	0 (0, 0.06), < 0.001

The content of this supplementary information has not been edited. All risk and liability rest with the authors.

Table S2. The estimated incidence risk ratios (IRR) (95% confidence intervals) for climate indices on the combined number of leptospirosis cases in cattle across four consecutive seasons per district in New Zealand by Poisson regression models, accounting for season, number of farms, and North/South island as fixed effects and district as a random effect.

		Frequency of extreme rainfall (days/season)	Seasonal mean rainfall (mm)	Frequency of surface runoff (days/season)	Seasonal mean soil moisture (mm)
Intercept	Intercept, p-value	−2.41, < 0.001	−2.49, < 0.001	−2.43, < 0.001	−2.13, < 0.001
Climate Variable	IRR, p-value	1.23 (0.57, 2.63), 0.6	1.03 (0.98, 1.08), 0.28	1.03 (1, 1.06), 0.08	1 (1, 1.01), 0.07
Season: Summer	IRR, p-value	Reference			
Season: Autumn	IRR, p-value	1 (0.83, 1.21), 1	0.98 (0.81, 1.19), 0.84	0.92 (0.75, 1.14), 0.45	0.89 (0.71, 1.12), 0.33
Season: Winter	IRR, p-value	0.97 (0.8, 1.17), 0.75	0.93 (0.76, 1.13), 0.46	0.71 (0.48, 1.05), 0.09	0.74 (0.52, 1.04), 0.08
Season: Spring	IRR, p-value	1.03 (0.85, 1.25), 0.75	1.01 (0.83, 1.22), 0.95	0.93 (0.75, 1.16), 0.52	0.85 (0.65, 1.12), 0.25
Farm count: Dairy	IRR, p-value	1.17 (0.67, 2.03), 0.59	1.16 (0.67, 2.02), 0.6	1.14 (0.66, 1.99), 0.64	1.15 (0.66, 2), 0.62
Farm count: Beef	IRR, p-value	2.21 (1.27, 3.85), 0.01	2.22 (1.27, 3.88), < 0.001	2.25 (1.29, 3.93), < 0.001	2.23 (1.28, 3.9), < 0.001
Island: North	IRR, p-value	Reference			
Island: South	IRR, p-value	0.22 (0.07, 0.63), < 0.001	0.22 (0.07, 0.64), 0.01	0.22 (0.07, 0.63), < 0.001	0.22 (0.08, 0.64), 0.01

The content of this supplementary information has not been edited. All risk and liability rest with the authors.
